# Genome-wide association mapping and candidate genes analysis of high-throughput image descriptors for wheat frost tolerance

**DOI:** 10.1007/s44154-025-00257-2

**Published:** 2025-12-10

**Authors:** Rui Yu, Yixue Liu, Meng Yuan, Pingtao Jiang, Jiwen Zhao, Chuanliang Zhang, Xiaowan Xu, Qilin Wang, Yuze Wang, Tiantian Chen, Jingrui Ou, Yihang Luo, Haitao Dong, Zhensheng Kang, Qingdong Zeng, Yusheng Zhao, Shouyang Liu, Baofeng Su, Dejun Han, Jianhui Wu

**Affiliations:** 1 College of Agronomy, Northwest A&F University, Yangling, 712100 Shaanxi China; 2 Present Address: State Key Laboratory for Crop Stress Resistance and High-Efficiency Production, Northwest A&F University, Yangling, 712100 Shaanxi China; 3 Present Address: College of Mechanical and Electronic Engineering, Northwest A&F University, Yangling, 712100 Shaanxi China; 4 Institute of Crop Sciences, Chinese Academy of Agricultural Sciences (CAAS), Beijing, 100081 China; 5 College of Plant Protection, Northwest A&F University, Yangling, 712100 Shaanxi China; 6 Institute of Genetics and Developmental Biology, Chinese Academy of Sciences (CAS), Beijing, 100101 China; 7 Engineering Research Center of Plant Phenotyping, Ministry of Education, Academy for Advanced Interdisciplinary Studies, Nanjing Agricultural University, Nanjing, 210095 Jiangsu China

**Keywords:** Wheat, Frost, Image, GWAS (genome-wide association study), GS (genomic selection)

## Abstract

Repeated occurrences of extreme weather events, such as low temperatures, due to global warming present a serious risk to the safety of wheat production. Quantitative assessment of frost damage can facilitate the analysis of key genetic factors related to wheat tolerance to abiotic stress. We collected 491 wheat accessions and selected four image-based descriptors (BLUE band, RED band, NDVI, and GNDVI) to quantitatively assess their frost damage. Image descriptors can complement the visual estimation of frost damage. Combined with genome-wide association study (GWAS), a total of 107 quantitative trait loci (QTL) (*r*^*2*^ ranging from 0.75% to 9.48%) were identified, including the well-known frost-resistant locus *Frost Resistance* (*FR*)*-A1*/ *Vernalization* (*VRN*)*-A1*. Additionally, through quantitative gene expression data and mutation experience verification experiments, we identified two other frost tolerance candidate genes *TraesCS2A03G1077800* and *TraesCS5B03G1008500*. Furthermore, when combined with genomic selection (GS), image-based descriptors can predict frost damage with high accuracy (*r* ≤ 0.84). In conclusion, our research confirms the accuracy of image-based high-throughput acquisition of frost damage, thereby supplementing the exploration of the genetic structure of frost tolerance in wheat within complex field environments.

**Supplementary Information:**

The online version contains supplementary material available at 10.1007/s44154-025-00257-2.

## Introduction

The increasing frequency of extreme weather represents a threat to global food security and poses a severe challenge in meeting future food demands (Mickelbart et al. 2015; Zhang et al. 2021). Frost, as a type of extreme weather, causes the most severe crop damage compared to other extreme conditions and has been frequently recorded in agricultural regions worldwide (Papagiannaki et al. 2014; Rezaei et al. 2023). It generally refers to temperatures below 0 °C, which promote the formation of ice crystals within plant cells for a certain period, leading to cell rupture and ultimately resulting in loss of production (Guo et al. 2018). Overwintering wheat, accounting for 75% of wheat yield and grown in higher latitude environments, is particularly susceptible to frost damage, with potential losses reaching up to 80% under the most severe conditions in the field (Soleimani et al. 2022; Xiao et al. 2022; Yue et al. 2016). Additionally, for winter wheat, the vernalization process and the response to cold occur simultaneously, with vernalization being achieved at a sustained low temperature (optimal at 4 °C) (Hassan et al. 2021). Different types of winter wheat, such as semi-winter and spring types, exhibit variations in vernalization requirements and temperature thresholds due to the presence of different vernalization gene allele types in each subgenome (Hyles et al. 2020). Consequently, their tolerance to frost damage during vernalization varies. Despite the cultivation of the most suitable types of winter wheat in different ecological regions, rapid and extensive damage occurs during frost events. Therefore, frost tolerance has become an important physiological target for wheat ecological adaptation breeding, urgently requiring the cultivation of winter wheat that is more tolerant to frost to cope with extreme climate stress and promote sustainable agricultural development (Hassan et al. 2021).

To mitigate the negative effects of frost on wheat production, it is necessary to identify quantitative trait loci (QTL) or genes that can improve wheat frost tolerance or winter survival. Previous genetic studies of frost tolerance in wheat have identified several crucial genetic factors (Mickelbart et al. 2015). Two major frost tolerance loci, *Frost Resistance* (*FR*)*-A1* and *FR*-*A2*, have been identified on chromosome 5A (Vágújfalvi et al. 2000; Zhu et al. 2014). *FR-A1*, located close to *Vernalization* (*VRN*)*-A1*, has been suggested to exhibit a pleiotropic effect of *VRN-A1*(Dhillon et al. (Dhillon et al. 2010). *FR-A2* has been identified approximately 30 cM away from *VRN-A1* and is defined as a region containing at least 11 copies of the *C-repeat Binding Factor* (*CBF*) gene (Vagujfalvi et al. 2003; Wurschum et al. 2017). Although*FR-A1* and *FR-A2* jointly regulate frost tolerance in wheat, the specific biological mechanisms remain unclear (Galiba et al. 2009; Zhu et al. 2014). Recently, QTL mapping and genome-wide association studies (GWAS) have revealed new frost tolerance hotspots in wheat's homologous groups 4 and 7, but these loci are not sufficient to meet improvement needs (Soleimani et al. 2022; Zhang et al. 2022; Zhao et al. 2020). Specifically, the multiple copy genes at the*FR-A2* locus, namely *VRN-A1*, *VRN-B3*, *PPD-B1*, and *PPD-D1*, have been demonstrated through GWAS to play a crucial role in wheat frost tolerance (Babben et al. 2018). However, only constructive suggestions were proposed for developing marker-assisted breeding. Additionally, there is only one well-defined cold signaling pathway, the*inducer of CBF expression* (*ICE*)-CBF cold response (COR) pathway (Liu et al. 2019; Shi et al. 2018), emphasizing the urgent need for further research on the genetics and molecular biology of wheat frost tolerance.

Rapid quantification of frost damage is paramount in understanding the extent of damage and planning for effective mitigation (Gobbett et al. 2021). When dealing with frost damage in extensive and diverse wheat production fields, traditional visual scoring for damage cannot be efficiently carried out (Furbank and Tester 2011). High-throughput phenotyping methods can facilitate the collection of large amounts of data within a short timeframe and have been utilized in assessing frost damage in forests; however, their application in evaluating wheat frost damage is limited (Araus et al. 2018).

This study aimed to utilize an unmanned aerial vehicle (UAV) and imaging to characterize wheat frost damage in the field. We assessed a wheat association panel comprising 491 genotypes under naturally occurring frost temperatures. NDVI, GNDVI, the BLUE band, and the RED band were extracted from high-throughput image analysis pipelines to gauge wheat's response to frost. Our research aimed to compare the efficacy of image-based phenotypic methods and traditional visual estimation in assessing frost phenotypes, alongside integrating GWAS to identify frost tolerance QTL and candidate genes. Furthermore, the accuracy of image-based features was validated using a genomic selection (GS) prediction model, and potential breeding markers were identified through single-nucleotide polymorphisms (SNPs). Our study lays the groundwork for analyzing the genetic structure of wheat abiotic stress in conjunction with high-throughput phenotyping methods.

## Results

### Frost tolerance of diverse panels at low temperatures

**Fig. 1:**
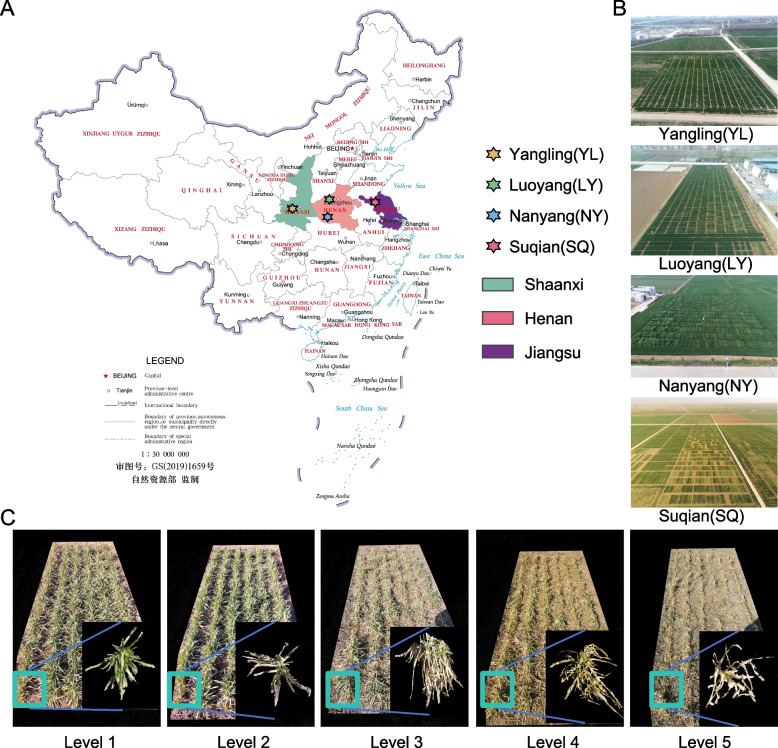
**A** Geographical locations of the four sites in China; **B** Locations of Luoyang (LY) and Nanyang (NY) in Henan province, Yangling (YL) in Shaanxi province, and Suqian (SQ) in Jiangsu province; **C** Visual representation of frost damage levels ranging from level 1 to 5, accompanied by detailed plant images corresponding to each level in the bottom right corner

**Fig. 2:**
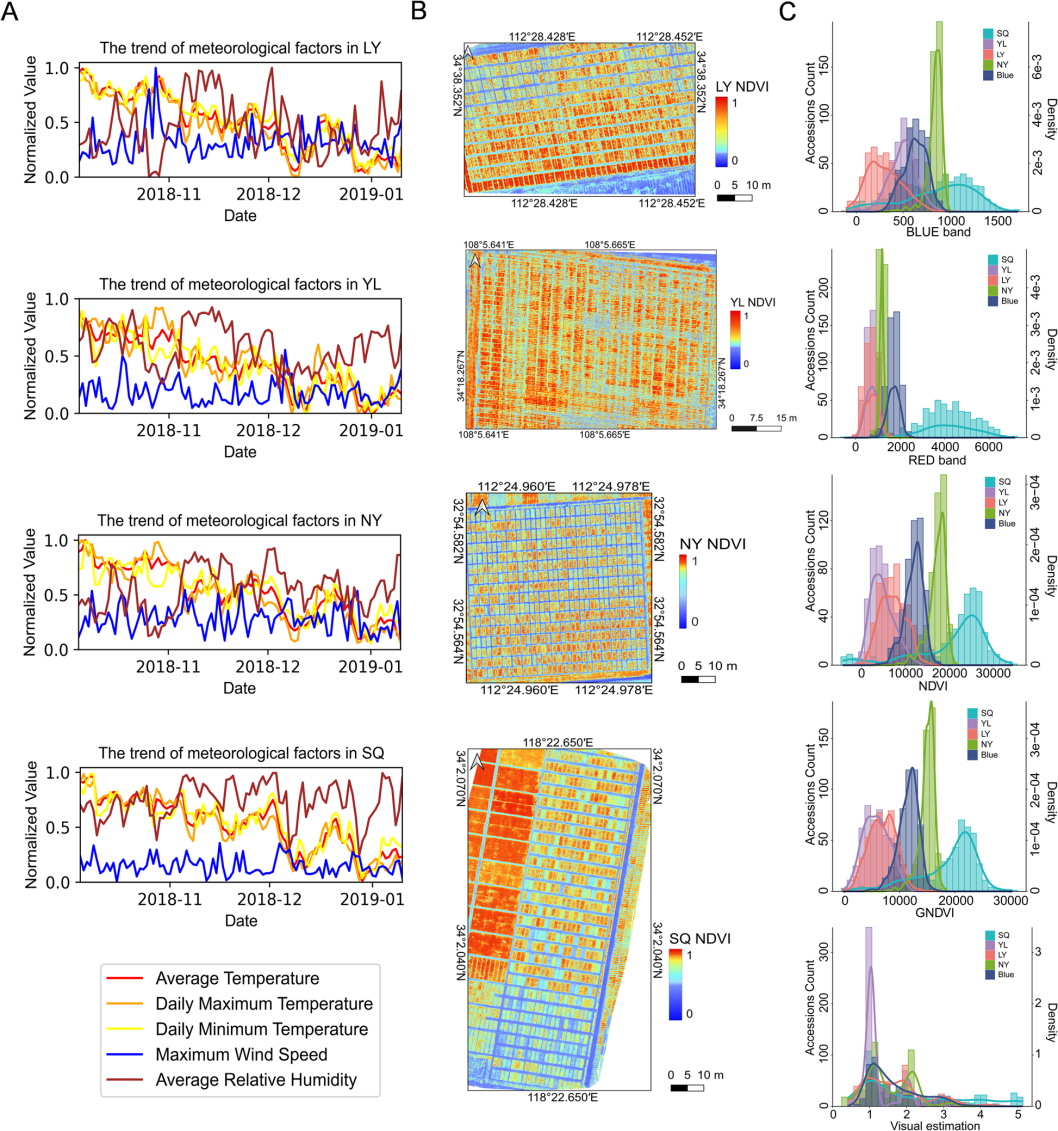
Comparison of weather factors, including BLUE, RED, NDVI, GNDVI, and canopy visual estimation of frost across four environments. **A** Normalized values of average temperature, daily maximum temperature, daily minimum temperature, maximum wind speed, and average relative humidity for each site from October 5, 2018, to January 10, 2019. The x-axis represents the day, and the y-axis represents the normalized value. Different colors of the lines indicate various weather factors as shown in the legend; **B** Pseudo-color images of NDVI for each site obtained through drone image processing. The x-axis and y-axis show the spatial coordinates of the images, while the color bar represents the NDVI values ranging from 0 to 1; **C** Histograms displaying the frequency distribution of phenotypic data for BLUE, RED, NDVI, GNDVI, and visual estimation across the four environments and Blue values

The genome-wide association mapping panel was cultivated at various locations in China: in Luoyang (LY) and Nanyang (NY) in Henan province; in Yangling (YL) in Shaanxi province; and in Suqian (SQ) in Jiangsu province (Fig. 1A). A total of 491 diverse genotypes were used in this study, all of which were cross-bred and released at different times from the major wheat-producing regions of China. In this section, we delineate the weather factors influencing wheat frost tolerance and growth across diverse environments. Comprehensive details regarding the experimental sites and corresponding weather data are provided in Table S4. Weather patterns manifest distinct trends across the four environments (Fig. 2A). YL notably exhibits minimal variation in weather factors, contrasting with SQ, which displays the most variability characterized by numerous inflection points in relative humidity and pronounced temperature drops. LY and NY demonstrated moderate fluctuations in weather conditions, with NY notably featuring higher humidity levels compared to the other sites.

In terms of phenotypic variations among the four environments (Fig. 2B and C), SQ displayed the most diverse phenotypes, including some extreme values (5), whereas YL did not exhibit any values of 4 or 5. Across all sites, most of the visual estimations of the phenotypes were 1 and 2. The phenotypic distribution related to frost damage, such as NDVI, followed an approximate normal distribution, highlighting the potential utility of UAV image descriptors for assessing wheat canopy status and identifying genes associated with frost tolerance. Density curves and frequency distribution histograms depicting central tendency and variability in canopy visual estimations across sites. The presence of outliers, particularly in SQ, underscores the significant impact of environmental factors on phenotype expression. These results suggest that weather conditions exert varying effects on wheat frost tolerance and growth across diverse environmental settings.

Correlation analysis revealed highly significant positive relationships (*p* < 0.01) among four UAV data remote across most environments. Moreover, at the Suqian by severe frost damage, these four UAV data exhibited a statistically significant negative correlation (*p* < 0.01) with visual estimation (Table S5). *H*^*2*^ of visual estimation, BLUE band, RED band, GNDVI, and NDVI were 0.51, 0.57, 0.12, 0.33, and 0.39, respectively. The heritability of the image-based frost tolerance trait was lower compared to that of visual evaluation, possibly due to the image-based descriptors captured phenotypic differences in wheat varieties influenced by varying weather conditions before and during frost events across different experimental sites. In contrast, visual scoring, with its coarse grading system, resulted in smaller phenotypic variations (Xiao et al. 2018).

### Genetic diversity of wheat panel

After filtering out low-quality SNP markers, a total of 410,556 SNP markers were retained for subsequent analyses. Of these, 403,928 markers were successfully mapped to specific positions on 21 chromosomes (Table S2). Genetic diversity evaluation using markers on chromosomes revealed that wheat populations displayed substantial genetic variation, with polymorphism information content (PIC) values averaging 0.28 (0.26–0.31) and heterozygosity (He) values averaging 0.70 (0.65–0.79) across all genomes. The population structure differentiation pattern, defining the number of subpopulations (K) ranging from 2 to 14 (Fig. S2; Fig. S3A), was consistent with discrete clusters in the principal component analysis (PCA) (Fig. S3B). For the optimal population structure (K = 4) (Fig. S2), Sp1 mainly consisted of Chinese landraces (CL); Sp2 predominantly included introduced modern cultivars (IMC); Sp3 mainly included Chinese spring cultivars (CSC); Sp4 predominantly included mixed winter cultivars (MWC). Sp3-Sp4 were combined as modern Chinese cultivars (MCC). Linkage disequilibrium (LD) analysis, using pairwise comparisons of 410,556 SNPs, showed that LD (*r*^2^) decayed to the critical value (0.1) at approximately 3.6 Mb for the whole genome. LD decay was fastest in the D genome at 1.6 Mb, followed by the A genome at 3 Mb, and the B genome at 5.7 Mb (Fig. S3C). The panel exhibited comparable genetic diversity and geographic representativeness to previously reported studies (Yu et al. 2024). The kinship matrix displayed population differentiation outcomes that resembled the population structure (Fig. S3D).

### Identification of frost tolerance QTL by GWAS

**Fig. 3:**
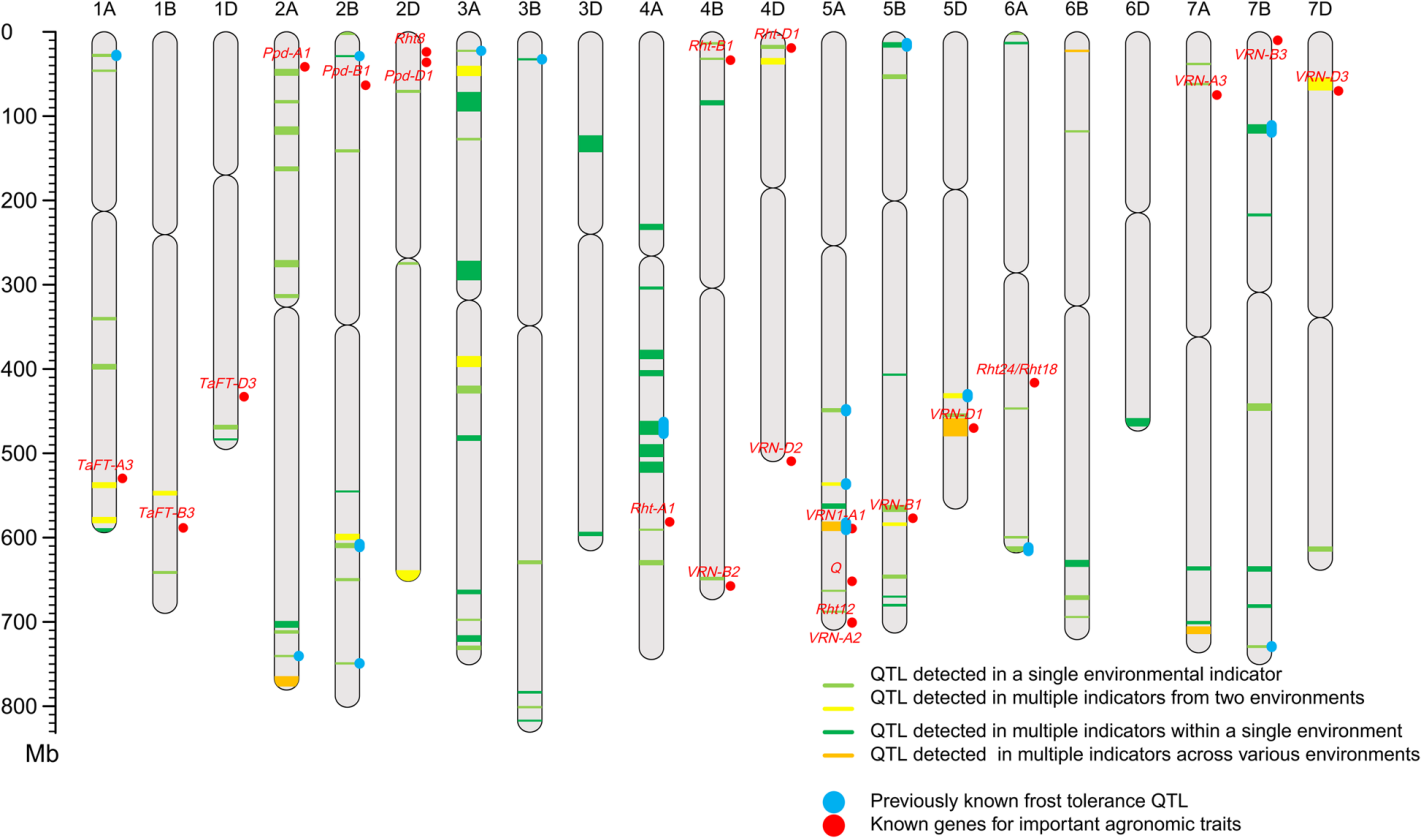
Distribution of frost tolerance QTL identified in this study and known frost tolerance QTL on wheat chromosomes. The blue and red points represent previously known frost tolerance QTL and known agronomically important genes, respectively. Light green bands denote frost tolerance QTL detected by GWAS in a single environmental indicator; dark green bands signify frost tolerance QTL discovered by GWAS in multiple indicators within a single environment; light yellow bands represent frost tolerance QTL detected by GWAS in multiple indicators from two environments; dark yellow bands represent frost tolerance QTL identified by GWAS in multiple indicators across various environments. The band widths reflect the confidence intervals

GWAS was performed for the frost tolerance phenotype across all environments and Blue. The Manhattan plots of the image descriptors and visual estimation are depicted in Fig. S4 and Fig. S5. In total, 107 QTL were identified across 21 chromosomes and explained phenotypic variances ranging from 0.75% to 9.48% (Fig. 3 and Table S6). Forty-two QTL were detected by manual visual estimation; 54, 39, 55, and 49 QTL were detected by BLUE band, RED band, NDVI, and GNDVI, respectively. Of the total QTL, 34 QTL were detected by a single Spectral Vegetation Index (SVI, eight of which overlap with Visual estimation); 18 QTL were identified by two SVIs (seven overlapped with Visual estimation); 17 QTL were detected simultaneously by three SVIs (six overlapping with Visual estimation); 19 QTL were consistently identified by all SVIs (two overlapping with Visual estimation). Additionally, another 19 QTLs were exclusively detected by Visual estimation. Seventeen QTL overlapped or were adjacent to previously studied wheat frost tolerance QTL, such as *QFr.nwafu-5A.2* overlapping with *LT-QTL33* and *qCT5A.5* (Pang et al. 2021); *QFr.nwafu-5A.4* overlapping with *QWs.ugw-5A.2* (Chen et al. 2019), *LT-QTL34* (Zhao et al. 2020), and *qCT5A.7* (Pang et al. 2021). Among them, *QFr.nwafu-5A.4* was located between 583.976191 and 592.850732 Mb of Chr5A, where the reported major locus *FR-A1*/*VRN-A1* for frost tolerance was identified (Sutka and Snape 1989), and *QFr.nwafu-5A.4* was associated with four indicators across all environments. To eliminate false association caused by admixture of spring and winter wheat materials in the panel, 194 winter wheat materials carrying the *vrn-A1* allele were selected for association analysis (Fig. S6). A significant peak persisted at *QFr.nwafu-5A.4*, suggesting that *FR-A1* is the candidate freezing tolerance locus in this region.

Additionally, a homologous gene *VRN-D1* of *VRN-A1* was found in *QFr.nwafu-5D.3*, which was associated with four indicators across all environments. Moreover, *QFr.nwafu-7D.1* was identified in two environments, within which *VRN-D3/FT-D1* has been reported. *VRN-D3*/*FT-D1*is known to regulate wheat flowering and low-temperature response pathways in Arabidopsis and wheat (Michaels and Amasino 1999; Yan et al. 2006).

### Identification of frost tolerance candidate genes

The identification of candidate genes primarily focused on the genes within QTL localized across multiple environments and indicators. Initially, *TraesCS2A03G1077800* encodes a calcium-dependent protein kinase and is located in *QFr.nwafu-2A.7*. It shares 91.95% protein identity with *OsCDPK7* in *Oryza sativa* (Saijo et al. 2000). In rice, *OsCDPK7* has been shown to mediate the activity of membrane channels and sugar metabolism, positively regulating frost tolerance (Saijo et al. 2001). Resequencing data identified 65 polymorphic DNA variants in the upstream, gene body, and downstream regions of *TraesCS2A03G1077800*. Among these, 27 markers showed significant association with the phenotype (Table S7). However, only the significant SNP *s2A_708549821* (T/C), located at 100 bp in the gene coding sequence, causes a missense mutation (Fig. S7A and B). The effects of Hap1 (CC) and Hap2 (TT) on visual estimation, GNDVI, NDVI, BLUE band, and RED band showed significant differences (Fig. S7C). Visual estimation of Hap1 was lower, while growth potential was higher compared to Hap2. Among over 2,000 worldwide germplasms, Hap1 was more abundant in winter-type and semi/weak winter-type varieties (51% and 29%, respectively), whereas spring-type varieties contained a higher proportion of Hap2 (38%) (Fig. S7D and Table S8). Additionally, this gene exhibited expression both preceding and following cold exposure in KN9204, but demonstrated significantly increased expression levels after frost treatment (Fig. S7E).

**Fig. 4:**
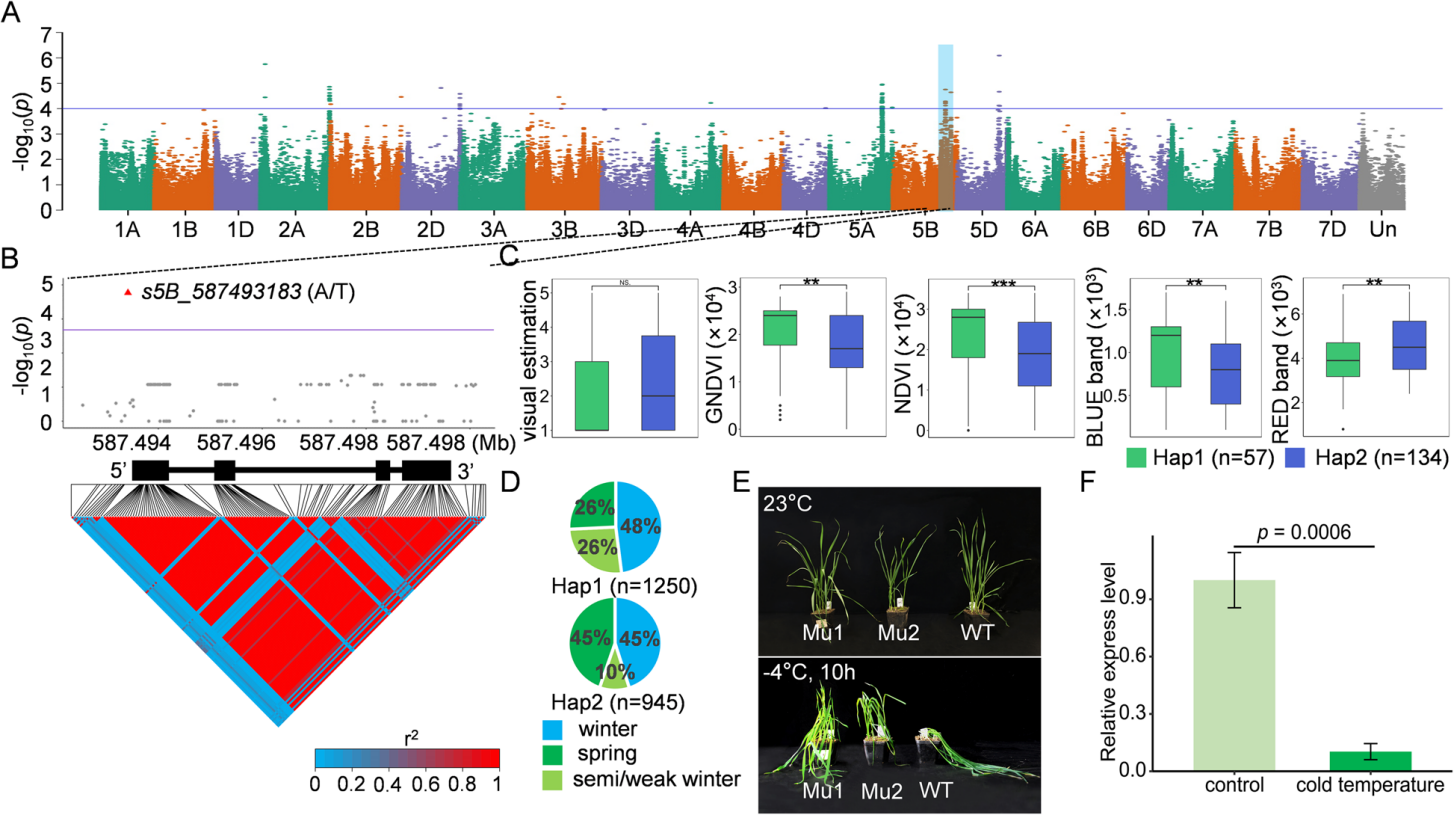
Variation in *TraesCS5B03G1008500*. **A** Manhattan map demonstrating visual estimation in NY, with gene loci highlighted in blue on chromosome 5B and significant variation shaded in purple (− log10[*P*-value] = 4); **B** Local Manhattan plot (top) and LD heat map (bottom) surrounding *TraesCS5B03G1008500*. The red color indicates strong LD with significant variation. Red triangles represent variation in *TraesCS5B03G1008500*; **C** Comparison of visual estimation, GNDVI, NDVI, BLUE band, and RED band for the two haplotypes in SQ. *p*-values were calculated using t-tests (*, *P* < 0.05; **, *P* < 0.01; ***, *P* < 0.001; NS, not significant); **D** Percentages of haplotypes in different types of wheat; **E** The upper panel displays the growth phenotypes of the two mutants and wild-type KN9204 at 23 °C, while the lower panel shows their growth status following a 10-h frost damage treatment at −4 °C; **F** Comparison of the differences in gene relative expression level between control (23 °C) and cold temperatures (−4 °C) for this gene in KN9204

In *QFr.nwafu-5B.5*, an upstream gene variant, *s5B_587493183* (A/T), was identified in *TraesCS5B03G1008500* (Fig. 4A and B). Beyond visual estimation, the effects of Hap1 (TT) and Hap2 (AA) on GNDVI, NDVI, BLUE band, and RED band showed significant differences (Fig. 4C). Among over 2,000 worldwide germplasms, Hap1 was more prevalent in winter-type and semi/weak winter-type varieties (48% and 26%, respectively), while spring varieties contained a higher proportion of Hap2 (35%) (Fig. 4D and Table S9). Both mutant lines (Mu1 and Mu2) exhibited significantly enhanced frost tolerance compared with the wild-type cultivar KN9204 (WT) (Fig. 4E). This gene exhibited expression both preceding and following cold exposure in KN9204, but demonstrated significantly reduced expression levels after frost treatment (Fig. 4F).

### Whole genome prediction of frost tolerance

**Fig. 5:**
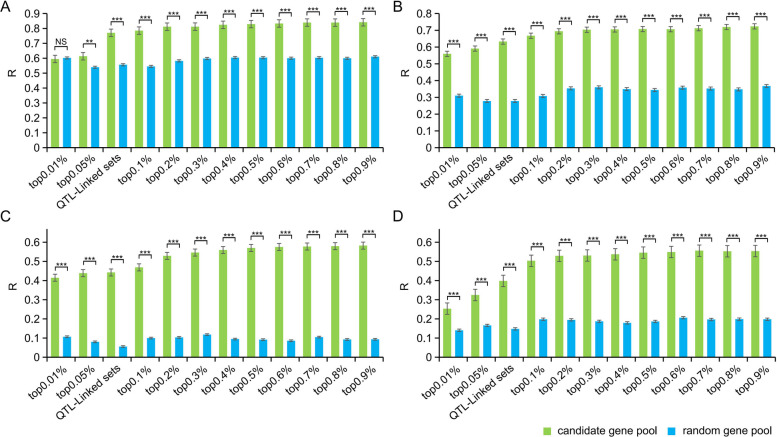
Prediction accuracies of different sets of SNPs in the candidate gene pool and random gene pool for frost tolerance related to GNDVI in SQ(**A**), LY(**B**), NY(**C**), and YL(**D**). GREEN represents the candidate gene pool, and BLUE signifies the random gene pool. Top 0.01%, top 0.05%, top 0.1%, top 0.2%, top 0.3%, top 0.4%, top 0.5%, top 0.6%, top 0.7%, top 0.8%, and top 0.9% refer to the top 0.01%, top 0.05%, top 0.1%, top 0.2%, top 0.3%, top 0.4%, top 0.5%, top 0.6%, top 0.7%, top 0.8%, and top 0.9% of the total SNPs extracted according to low *p*-values from GWAS. QTL-linked SNP sets are based on significant *p*-values from GWAS. Significance was determined using t-tests (*, *P* < 0.05; **, *P* < 0.01; ***, *P* < 0.001; NS, not significant)

GS analyses were conducted using RR-BLUP to determine the accuracy of specific combinations in selecting for frost tolerance-related phenotypes in winter. Initially, the prediction accuracies of both the candidate gene pool and the random gene pool increased dramatically with the number of SNPs and then gradually plateaued. The prediction accuracy of SNPs from different sets of the candidate gene pool was significantly higher than that of the random gene pool, with QTL SNP sets exhibiting good prediction accuracy across four different environments. The prediction accuracies of the candidate gene pool in SQ, LY, NY, and YL ranged from 0.53 to 0.87, 0.40 to 0.80, 0.04 to 0.61, and 0.12 to 0.70, respectively (Table S10). This indicates that SNP sets from the candidate gene pool, including QTL-linked sets, could be potential biomarkers for frost tolerance during selection and breeding. In the candidate gene pool, the SNP sets that reach the plateau were not limited to QTL-linked sets; most prediction plateaus started at the top 0.3%. Certain SNP sets that exceeded QTL-linked sets showed higher prediction accuracy than QTL-linked sets. For example, the prediction accuracy of GNDVI in four environments by the QTL-linked sets was lower than that of the prediction plateau (top 0.3%), ranging from 0.04 to 0.13 (Fig. 5). This suggests that many minor-effect loci, which went undetected in GWAS, still contribute to the overall performance of frost tolerance, thereby increasing accuracy when markers associated with these minor-effect loci are included in the prediction model (Pang et al. 2021).

## Discussions

### Image based assessment of frost damage in diverse panels

One of the primary challenges in enhancing wheat frost tolerance is the genotype by environment interaction (G × E), which implies that different genotypes respond differently to environmental variation. Environmental variation includes weather factors such as temperature, humidity, and wind speed, all of which influence the occurrence and severity of frost damage (Gobbett et al. 2021; Xiao et al. 2018). In our study, we observed significant phenotypic differences among the four sites (LY, YL, NY, and SQ) regarding both visual and image-based frost tolerance scores. These differences primarily arose from varying degrees of frost damage and different growth stages of the wheat plants at each site. For instance, SQ experienced the highest accumulated temperature before frost damage, promoting vegetative growth and rendering the plants more susceptible to low temperatures (Stutsel et al. 2020). This explained why the wheat in SQ suffered more severe frost damage despite exhibiting high values of vegetation indices and single bands. Conversely, YL had the lowest accumulated temperature and the least phenotypic variation. This disparity may stem from an extended exposure to low temperatures compared to the other experimental sites, leading to an early saturation of vernalization in wheat, a cold-tolerant process crucial for wheat development.

Image descriptors are measurable characteristics of the wheat canopy derived from spectral images captured by UAVs. These descriptors differ from traditional phenotypic traits, such as visual estimation, in terms of accuracy, efficiency, and objectivity (Sarić et al. 2022). Image traits capture variations in wheat canopy reflectance characteristics influenced by frost stress, including leaf chlorophyll content, greenness, biomass, and canopy structure. Utilizing image traits as phenotypic data helps reduce noise and bias caused by environmental and human factors, thereby enhancing the power and resolution of GWAS. The image traits is significantly superior to the traditional method in accuracy and objectivity, but the latter is still of practical value in field rapid screening. Results from the chi-square test and feature selection indicated that RED, NDVI, BLUE, and GNDVI were the most important SVIs for predicting wheat frost tolerance across the four sites. This aligns with our hypothesis that these SVIs effectively capture relevant information about wheat canopy reflectance characteristics affected by frost stress. Previous studies have reported that RED bands are associated with leaf chlorophyll content, which may decrease due to frost damage (Martino and Abbate 2019), while NDVI and GNDVI serve as indicators of wheat plant greenness and biomass, both of which may decline due to frost injury (Arshad et al. 2023; Yin et al. 2023). Obtaining vegetation information in the BLUE band is challenging due to interference from various factors, and the relationship between its reflectivity and vegetation physiological characteristics (such as chlorophyll content, biomass, moisture content, etc.) is not evident (Xue et al. 2022). Nevertheless, this does not imply that the BLUE band lacks value; it may provide unique information in certain situations where other bands cannot (Hennessy et al. 2020). Based on the heritability of the image phenotype, the correlation with visual estimation and the results of association analysis, we believe that BLUE is the best indicator to predict the frost resistance of wheat. It may reflect the changes of wheat leaves after freezing damage, and it may also combine with other bands to form an effective VI. However, further research and analysis are needed to confirm this. The evaluation of the frost resistance of wheat still needs to integrate multiple indexes. By prioritizing the importance of SVIs, we can select the most informative features for constructing a predictive model while understanding the underlying mechanisms of wheat frost tolerance. Moreover, our results demonstrated the potential of using image descriptors derived from UAV data as a novel and efficient approach to evaluate wheat performance and identify frost-tolerant genes.

Moreover, the heritability of the image-based frost tolerance phenotype was found to be lower than that of visual estimation. Consideration the varying degrees of frost damage across the four environments, the heritability of image-based frost resistance traits was lower than that of visual estimation, reflecting environmental variability, phenotypic precision of the imaging system, and temporal discrepancies in frost occurrence. This disparity primarily arose from varying degrees of frost damage observed across the four environments. Visual estimation readily grouped materials with similar frost damage into the same grade, whereas image-based data could discern finer differences and unrelated phenotypic variations (Chen et al. 2019). Additionally, temporal variations in frost occurrence across different environments contributed to this discrepancy. Despite efforts to synchronize data collection, observed phenotypic differences among wheat genotypes were attributed to their distinct adaptability to environmental conditions rather than disparities in growth stages post-frost.

### QTL for controlling frost tolerance

By combining GWAS with image-based frost tolerance phenotypes (VIs), this study successfully identified frost tolerance QTL that are partly linked to phenological development. The VI-based frost tolerance phenotypes revealed a greater number of QTL than those identified solely through visual estimation. Most QTL can be identified through both image-based frost tolerance phenotypes and visual estimation, while some are located using only one method. For instance, the well-known frost tolerance locus *FR-A1* was identified using both visual scores and image-based frost tolerance phenotypes, and *VRN-D3* was also located using visual estimation and image-based frost tolerance phenotypes. These results indicate the role of vernalization genes in the frost tolerance characteristics of wheat. Additionally, they demonstrate that image-based frost tolerance phenotype detection efficiency is similar to visual estimation, thus compensating for the limitations of visual estimation. For example, the image-based QTL for winter wheat survival rate localization effectively complements the localization efficiency of visual scores (Chen et al. 2019). Similarly, for other agronomic traits such as plant height, image-based QTL mining had a similar complementary effect on visual scores (Gao et al. 2023; Li et al. 2020; Wang et al. 2023; Wu et al. 2021).

The *FR-A1* locus, examined across multiple environments and various indices, is considered a candidate gene. *VRN-A1* and *FR-A1* on chromosome 5 A are tightly linked but physically separated. Different haplotypes in the *VRN-A1/FR-A1* segment cause significant variations in winter wheat cold resistance, and this segment has been utilized by breeders in cold resistance breeding (Kobayashi et al. 2005). Galiba et al (Galiba et al. 1995) mapped two QTL regulating vernalization requirement (*VRN-A1*) and frost tolerance (*FR-A1*) with a genetic distance of approximately 2 cM using a Chinese Spring 5A chromosome substitution line population. This finding was further validated by Kobayashi et al. through near-isogenic line analysis, confirming that *VRN-A1* and *FR-A1* diverged functionally by regulating *WAP1* (vernalization-related) and the *Wcbf2-Cor/Lea*pathway (frost tolerance-related), respectively (Kobayashi et al. 2005). Association analysis based on a panel of 194 winter wheat accessions carrying the*vrn-A1* allele was consistent with these results. However, another important frost tolerance locus, *FR-A2*, was not identified in this work. Examination of the 660 K genotype of this locus revealed minimal key mutations and a lack of polymorphism. Another possibility is that an invalid association owing to allelic heterogeneity. In standard GWAS, phenotypic distributions are analyzed independently at each nucleotide polymorphism site. Therefore, statistical significance is challenging to attain for genes with allelic heterogeneity, as allele-specific polymorphisms are compared with all other alleles (including null and functional variants) (Yano et al. 2016). After using resequencing data to increase markers, the *FR-A2* in the studied population could be divided into eight haplotypes (null haplotypes: *Hap A*_*1*_–*Hap A*_*4*_; functional haplotypes: *Hap B*_*1*_–*Hap B*_*4*_) (Fig. S8). Therefore, this may be the reason why *FR-A2* was not detected in this study, which can be further explored by gene-based association analysis in the future. Additionally, some significant frost tolerance QTL hotspots on 5 A, 5B, and 7B were also identified in this study.

### Exploring the frost tolerance candidate genes

In the domestication process of hexaploid winter wheat, cold acclimation and the vernalization requirement are essential for enabling winter wheat to thrive in a low-temperature environment, and specific genes related to these processes have been gradually selected (Xiao et al. 2018). Wheat frost tolerance is a complex trait controlled by multiple genes (Guo et al. 2018). In addition to CBF transcription factors, COR genes, and vernalization genes, other genes play crucial roles in regulating frost tolerance, such as *PAP6-like* (Zhang and Huo 2016), *Hsp90* (Li et al. 2019), *REP14* (Zhang et al. 2017), *TaPGK* (Zhang et al. 2023), and various transcription factors. In this study, along with *FR-A1/VRN-A1* and *VRN-D3*, we identified a homologous gene of rice *OsCDPK7*, which encodes a calcium-dependent protein kinase (Saijo et al. 2001). In cold signaling, Ca^2+^acts as a secondary messengers of temperature sensing, triggered by cold sensors located on membranes, leading to the activation of kinases that ultimately modulate the activity of ICE1 (Guo et al. 2018; Zhang et al. 2021). The Ca^2+^-dependent protein kinases (CPKs; also known as CDPKs) act as target sensors that can phosphorylate ABA-response effectors (Kidokoro et al. 2022). In rice, *OsCDPK7* is under stringent post-translational control in cells and has been shown to positively regulate frost tolerance (Saijo et al. 2001). *TraesCS2A03G1077800* exhibited high homology to *OsCDPK7* at the protein level (92%). We identified this candidate gene using wheat genetics, and further gene characterization requires validation through biological experiments. Additionally, *TraesCS5B03G1008500*, which encodes the cold acclimation protein, plays a role in protecting cell structure under low-temperature stress and directly participates in the downstream frost tolerance response. It has been chosen as a candidate gene for further analysis.

The transcriptome expression data of the three candidate genes showed significant differences compared to the control, thereby reinforcing the credibility of these candidate genes. Additionally, the high proportion of frost tolerance haplotypes in winter-type varieties further supports this perspective.

### GS assisted wheat breeding

Exploring various wheat frost tolerance genes, effective QTL, or key SNPs is essential for aiding in breeding (Michel et al. 2019). In this study, different sets of SNPs were utilized based on *p*-values from GWAS and specific QTL-linked SNP sets to conduct GS for various frost tolerance indices across different environments. The prediction accuracy of the top 0.3% of SNP sets, based on the *p*-values from GWAS, was higher than that based on QTL-linked SNP sets, regardless of whether the analysis involved visual estimation, vegetation index, or single bands, indicating that minor-effect SNPs have cumulative effects on frost tolerance (Chen et al. 2019). Across four environments, the prediction accuracy of SNP sets within candidate gene banks was significantly higher than that of a random library. The prediction accuracy of SNP sets based on*p*-values from visual evaluation in GWAS was comparable to that of SNP sets based on *p*-values from spectral indices and single-band GWAS. This demonstrated the accuracy of wheat frost damage phenotype acquisition and also validated that image indices could compensate for or even surpass traditional visual scores for phenotype acquisition and GS (Wu et al. 2021). This study provided a foundation for accelerating the breeding process of target organisms by utilizing GWAS or GS to analyze based on high-resolution genotypes and high-throughput phenotypes.

## Conclusion

This study employed visual estimation and image descriptors to evaluate frost damage in wheat across various environments. The results demonstrated that single bands (RED and BLUE) and vegetation indices (GNDVI and NDVI) effectively complemented visual estimation. Through the integration of GWAS and genetic analysis, QTL and genes associated with frost tolerance were identified. Furthermore, by incorporating GS, SNPs derived from image feature mining exhibited high prediction efficiency for frost damage phenotypes, establishing SNPs as viable biomarkers for wheat genetic enhancement. This research advanced the understanding of wheat frost tolerance-related genetic breeding by integrating high-throughput phenotyping and genotyping across multiple field environments.

## Materials and methods

### Plant materials

The genome-wide association mapping panel used in this study consisted of 491 diverse genotypes, included 49 Chinese landraces (CL), 86 introduced modern cultivars (IMC), and 356 modern Chinese cultivars (MCC). Among these, 122 were winter types, 198 were semi-winter types, 103 were weak winter types, and 68 were spring types. These cultivars were cross-derived and released at different times from the main wheat region in China. Detailed descriptions of the accessions are provided in Table S1. The second panel comprised 2,232 available genome re-sequences based on published data, accessible at http://wheatomics.sdau.edu.cn/. This second panel was primarily employed for candidate gene-based association analysis and haplotype distribution analysis.

### Field experimentation

The genome-wide association mapping panel was cultivated at various locations in China: in Luoyang (LY) (34.70°N, 112.50°E) on October 11, 2018, and in Nanyang (NY) (33.03°N, 112.50°E) on October 17, 2018, in Henan province; in Yangling (YL) (34.16°N, 108.40°E) on October 7, 2018, in Shaanxi province; and in Suqian (SQ) (34.02°N, 118.33°E) on October 12, 2018, in Jiangsu province (Fig. 1A). All experiments were organized using an augmented design with 14 blocks, and five controls were repeated in each block following ACBD-R (Burgueño 2018). Each plot measured 3.6 m^2^ with dimensions of 1.2 m × 3 m at a row spacing of 20 cm, and the plant density was maintained at 2.7 million per hectare across all experimental sites (Fig. 1B). Planting and management practices adhered to local guidelines. Micro meteorological stations were established at each experimental site to record the highest and lowest temperatures during the wheat growing season. On January 15, 2019, a significant cold wave swept across northern China, leading to extended periods where temperatures remained continuously below 0 ℃ in all experimental sites.

### Visually estimated data

At the time of wheat frost damage assessment, the wheat at the four experimental sites was at the overwintering stage (Feekes 3). Visual estimates and UAV data collection were carried out on wheat materials in YL, LY, NY, and SQ on January 19th, 20th, 21st, and 22nd, 2019, respectively. Visual estimates were based on the degree of frost damage to leaves and stems, categorized into 5 levels: (1) No part of the leaves succumbed to frost; (2) The frozen dead portion of the leaves was confined to the leaf tip, with minimal or no damage to lower leaves, and a majority of green leaves across the field; (3) The frozen dead portion of the leaves was less than the green leaf portion, but there was more damage to lower leaves, resulting in yellow leaves on the ground; (4) The frozen dead portion of the leaves exceeded the green leaf portion, with entirely dead leaves visible on the ground and fewer green leaves; (5) Both upper and lower leaves were completely frozen and yellow, with all leaves dead, potentially leading to the entire plant's demise. The actual situations are depicted in Fig. 1C.

### UAV data collection

Using the UAV for data collection while conducting visual estimation, we employed a UAV imaging system to capture multispectral images of the wheat canopy, offering high-resolution and timely information on crop phenotypes and stress responses. The system comprised a Matrice 100 drone equipped with a MicaSense camera featuring five narrowband spectral imagers. Reflectance calibration using the MicaSense whiteboard was conducted before and after each flight mission. The flights were conducted at an altitude of 15 m, scheduled between 12:30 and 14:30 on the same day to ensure consistent lighting conditions, and covered the entire experimental area with a substantial overlap (90%).

### Spectral Vegetation Indexes (SVIs) for wheat frost tolerance

Upon collecting and calibrating the UAV images, we utilized Pix4D Mapper 4.5.6 for processing multispectral images obtained from four experimental sites (YL, LY, NY, and SQ). Our processing workflow included geometric correction, radiation correction, image stitching, and index calculation to produce SVI maps corresponding to each site. SVIs were derived from UAV data collected using five narrowband spectral imagers: BLUE (475 nm, 20 nm), GREEN (560 nm, 20 nm), RED (668 nm, 10 nm), Red edge (717 nm, 10 nm), and NIR (840 nm, 40 nm). These specific spectral bands were selected for their sensitivity to wheat canopy reflectance properties, including leaf chlorophyll content, water content, and biomass (Raper and Varco 2014). SVI formulas were either adopted from existing literature or modified to suit our specific research objectives. Table1 provides details on the 10 SVIs employed in this study, including their formulas, categories, and references.

**Table 1:** Spectral vegetation indices and formulas

NO	SVI	Formula	References
1	BLUE	*R*_*b*_	-
2	GREEN	*R*_*g*_	-
3	RED	*R*_*r*_	-
4	Red edge	*R*_*re*_	-
5	NIR	*R*_*nir*_	-
6	NDVI	(*R*_*n*__*i*__*r*_-*R*_*r*_)/(*R*_*nir*_+*R*_*r*_)	(Thompson et al. 2019)
7	NDRE	(*R*_*nir*_-*R*_*re*_)/(*R*_*nir*_+*R*_*re*_)	(Maccioni et al. 2001)
8	GNDVI	(*R*_*nir*_-*R*_*g*_)/(*R*_*nir*_+*R*_*g*_)	(Gitelson et al. 1996)
9	CVI	(*R*_*nir*_×*R*_*r*_)/*R*_*g*_^2^	(Wu et al. 2008)
10	TVI	0.5×[120×(*R*_*nir*_-*R*_*g*_)-200×(*R*_*r*_-*R*_*g*_)]	(Broge and Leblanc 2001)

${R}_{b}$, ${R}_{g}$, ${R}_{r}$, ${R}_{re}$, ${R}_{nir}$ represent the gray value of BLUE, GREEN, RED, Red edge, and NIR orthophoto respectively

### Phenotypic data analysis

The phenotypic data comprised visually estimated frost damage and spectral vegetation indices (SVIs) strongly correlated with frost damage. To identify the key SVIs (Red, NDVI, BLUE, and GNDVI) crucial for wheat frost tolerance, a Python script was employed for feature selection based on the chi-square test (Fig. S1). This method evaluated the relationship between each SVI and the phenotypic trait, assigning scores to each SVI across different environments based on their relevance and importance for wheat frost tolerance. Additionally, phenotypic data derived from visually estimated frost tolerance and the image descriptors highly correlated with frost tolerance were used. Using the lme4 package in the R program (Bates et al. 2015), the best linear unbiased estimate (Blue) was estimated in a linear mixed model. Genotype and environments were treated as random effects in this model. The broad-sense heritability (*H*^2^) of all indices was calculated across the environments using the lme4 package in R 3.5.3, following formula Eq.1.

1 $${H}^{2}=\frac{{\sigma }_{g}^{2}}{{\sigma }_{g}^{2}+{\sigma }_{e}^{2}/{n}_{env}}$$

where ${\sigma }_{g}^{2}$ represents the variances of genotype, ${\sigma }_{e}^{2}$ represents the variances of error, ${n}_{env}$ represents the number of environments. Difference test was calculated using *t*-tests (*, *P* < 0.05; **, *P* < 0.01; ***, *P* < 0.001; NS, not significant).

### Genotyping, SNP filtering and population structure analysis

Leaf samples from 491 wheat accessions were gathered, and DNA was subsequently extracted. Subsequently, Wheat660K, the Affymetrix® Axiom® Wheat660 was employed to genotype the materials at the Beijing CapitalBio Technology Company (http://www.capitalbiotech.com). The polyploid version of the Affymetrix Genotyping Console (GTC) software was utilized for SNP genotype calling and allele clustering. SNP marker filtering criteria followed those outlined in Yu’s study (Yu et al. 2020), which included excluding markers with minor allele frequencies (MAF) < 0.05, missing data > 10%, and Hardy–Weinberg equilibria (HWE) > 0.01. Based on the formulas described by (Botstein et al. 1980)and (Nei 1978)respectively, two genetic diversity parameters, namely polymorphism information content (PIC) and expected heterozygosity (He), were calculated for each SNP marker and each chromosome. Population structure analysis, principal component analysis (PCA), construction of relative K-matrices, and genome-wide linkage disequilibrium (LD) analyses were performed as described in Wu’s methodology (Wu et al. 2020).

### Genome‑wide association studies

GWAS was conducted using a univariate linear mixed model in GEMMA software (Zhou 2012). Based on the modified Bonferroni correction, the suggested threshold was *P*= 1/Ne (where Ne = effective SNP number) (Li et al. 2012). Our results indicated that the suggestive*P*-value threshold for each chromosome ranged from 5.76 × 10^−4^ to 1.32 × 10^−4^ (Table S2). Therefore, we considered the mean value of 2.60 × 10^–4^ as the threshold for genome-wide significance. Manhattan plots and quantile–quantile plot (QQ plot) were generated using the qqman package in R 3.0.3 to visualize significant markers and the distribution of significant *P*-values in the GWAS results, respectively. The GCTA software was used to estimate the phenotypic variance explained (*r*^2^) by significant SNPs. Continuous significant markers within a 5 Mb distance were considered as a QTL. Identified GWAS loci were compared with previously reported QTL based on their physical locations in the Chinese Spring reference genome v2.1. For previously reported SG genes/QTL, the closest flanking markers were used to generate confidence intervals. The novelty of the identified GWAS loci depended on whether their QTL confidence intervals overlapped with reported QTL intervals.

### Candidate gene identification and quantitative RT-PCR analysis

Based on IWGSC RefSeq v2.1 gene annotations (http://wheatomics.sdau.edu.cn/), high confidence (HC) genes located within the QTL region around significant SNPs were selected for candidate gene analysis. Candidate gene-based association analysis was conducted using GAPIT 3.0, with a significance threshold set at *P* = 2.60 × 10^–4^. Additionally, total RNAs were extracted from the leaves of KN9204 using TRIzol reagent and were reverse transcribed into cDNA with FastKing Kit reverse transcriptase (TIANGEN). In turn, the expression levels of target genes was conducted on an QuantStudio3 real-time PCR system using a SYBR Premix Ex Taq Kit. The GhHistone3 gene (GenBank accession no. XM_044514280) was employed as an internal control. Relative changes in gene expression were estimated via the 2^−ΔΔCT^ method. Primers for qRT-PCR are listed in Table S3.

### GS analysis

First, the whole genome genes in wheat (SNPs) were divided into the candidate gene pool and the random gene pool, excluding QTL-linked SNP sets. Second, within the candidate gene pool, various SNP sets—top 0.01%, top 0.05%, top 0.1%, top 0.2%, top 0.3%, top 0.4%, top 0.5%, top 0.6%, top 0.7%, top 0.8%, and top 0.9%—based on the *p*-values from GWAS for each indicator under different environments, along with QTL-linked SNP sets based on significant *p*-values from GWAS for each indicator under different environments, were employed for GS. For the random gene pool, SNPs equating to the number of candidate gene pool were randomly selected each time, and this process was repeated 10 times. Subsequently, after obtaining all SNPs, the R package RR-BLUP (http://www.r-project.org/) (Endelman 2011) was utilized to predict the frost damage phenotype, with 50% used for training and 50% for testing. In this process, SNP sets from the candidate gene pool were repeated 500 times, while SNP sets from the random gene pool were also repeated 500 times. The final comparative results were based on 500 repeats of GS analyses for each given number of SNP sets. The correlation coefficient "r" between the predicted value and the observed value was used to assess the accuracy of the prediction.

## Supplementary Information

Supplementary Material 1: Table S1 Summary of the 491 worldwide wheat accessions. Table S2 The information of SNP markers on wheat chromosome. Table S3 List of the primers for quantitative real-time PCR in this study. Table S4 The information about experimental sites and corresponding environment. Table S5 Correlation analyses among different environments for visual estimation and UAV data. Table S6 Detailed information of all QTLs detected in each chromosome. Table S7 The significant DNA variants in the upstream, gene body, and downstream regions of *TraesCS2A03G1077800*. Table S8 Frost tolerance variations distribution in worldwide wheat accessions about *TraesCS2A03G1077800*. Table S9 Frost tolerance variations distribution in worldwide wheat accessions about *TraesCS5B03G1008500*. Table S10 Prediction accuracy of SNP sets for frost resistance phenotype.

Supplementary Material 2: Fig. S1 Chi-square test and feature selection of the spectral vegetation indices (SVIs) and the canopy visual estimation among four sites. Fig. S2 Estimated ΔK for structure analysis. Fig. S3 Genetic structure of the diverse genotypes. Fig. S4 Manhattan plots and Q-Q plots of BLUE, RED, NDVI, and GNDVI (from top to bottom) in SQ (A), LY (B), NY (C), and YL (D). Fig. S5 Manhattan plots and Q-Q plots for Visual estimation (A), BLUE band (B), RED band (C), NDVI (D), and GNDVI (E) in the best linear unbiased estimate (Blue). Fig. S6 Manhattan plots and Q-Q plots for Visual estimation (A), BLUE band (B), RED band (C), NDVI (D), and GNDVI (E) in the best linear unbiased estimate(Blue) of the 194 winter wheat materials carrying the *vrn-A1* allele. Fig. S7 Variation in *TraesCS2A03G1077800*. Fig. S8 The *FR-A2* haplotype is analyzed in this panel.

## Data Availability

The genotype and phenotype data presented in this study are available on reasonable request from the corresponding author. The data processing code presented in this study is available on the website https://github.com/yurui2024/Frost-tolerance.

## Abbreviations

Blue: Best linear unbiased estimate
CBF: C-repeat Binding Factor
CL: Chinese landraces
cM: Centimorgan
COR: Cold response
CSC: Chinese spring cultivars
FR: Frost resistance
FT: Flowering locust
GNDVI: Green normalized difference vegetation index
GS: Genome selection
GTC: Genotyping console
GWAS: Genome-wide association studies
H^2^: Heritability
Hap: Haplotype
HC: High confidence
HWE: Hardy–Weinberg equilibria
ICE: Inducer of CBF expression
IMC: Introduced modern cultivars
LD: Linkage disequilibrium
LY: Luoyang
MAF: Minor allele frequencies
MCC: Modern Chinese cultivars
MWC: Mixed winter cultivars
NDVI: Normalized difference vegetation index
Ne: Effective SNP number
NS: Not significant
NY: Nanyang
PCA: Principal component analysis
PPD: Photoperiod
QQ: Quantile–quantile
QTL: Quantitative trait loci
RNA: Ribonucleic Acid
RR-BLUP: Rirdge regression-Best linear unbiased prediction
SNP: Single-nucleotide polymorphism
SQ: Suqian
SVI: Spectral vegetation indexe
UAV: Unmanned aerial vehicle
VRN: Vernalization
YL: Yangling
