# Supplementary material for: Genome-wide association mapping and candidate genes analysis of high-throughput image descriptors for wheat frost tolerance
Source: Stress Biol. 2025 Dec 10;5(1):75. doi: 10.1007/s44154-025-00257-2 (PMC12690036; doi:10.1007/s44154-025-00257-2)
Supplement: Supplementary file 2 — Supplementary Material 2: Fig. S1 Chi-square test and feature selection of the spectral vegetation indices (SVIs) and the canopy visual estimation among four sites. Fig. S2 Estimated ΔK for structure analysis. Fig. S3 Genetic structure of the diverse genotypes. Fig. S4 Manhattan plots and Q-Q plots of BLUE, RED, NDVI, and GNDVI (from top to bottom) in SQ (A), LY (B), NY (C), and YL (D). Fig. S5 Manhattan plots and Q-Q plots for Visual estimation (A), BLUE band (B), RED band (C), NDVI (D), and GNDVI (E) in the best linear unbiased estimate (Blue). Fig. S6 Manhattan plots and Q-Q plots for Visual estimation (A), BLUE band (B), RED band (C), NDVI (D), and GNDVI (E) in the best linear unbiased estimate(Blue) of the 194 winter wheat materials carrying the vrn-A1 allele. Fig. S7 Variation in TraesCS2A03G1077800. Fig. S8 The FR-A2 haplotype is analyzed in this panel. [file 44154_2025_257_MOESM2_ESM.docx]

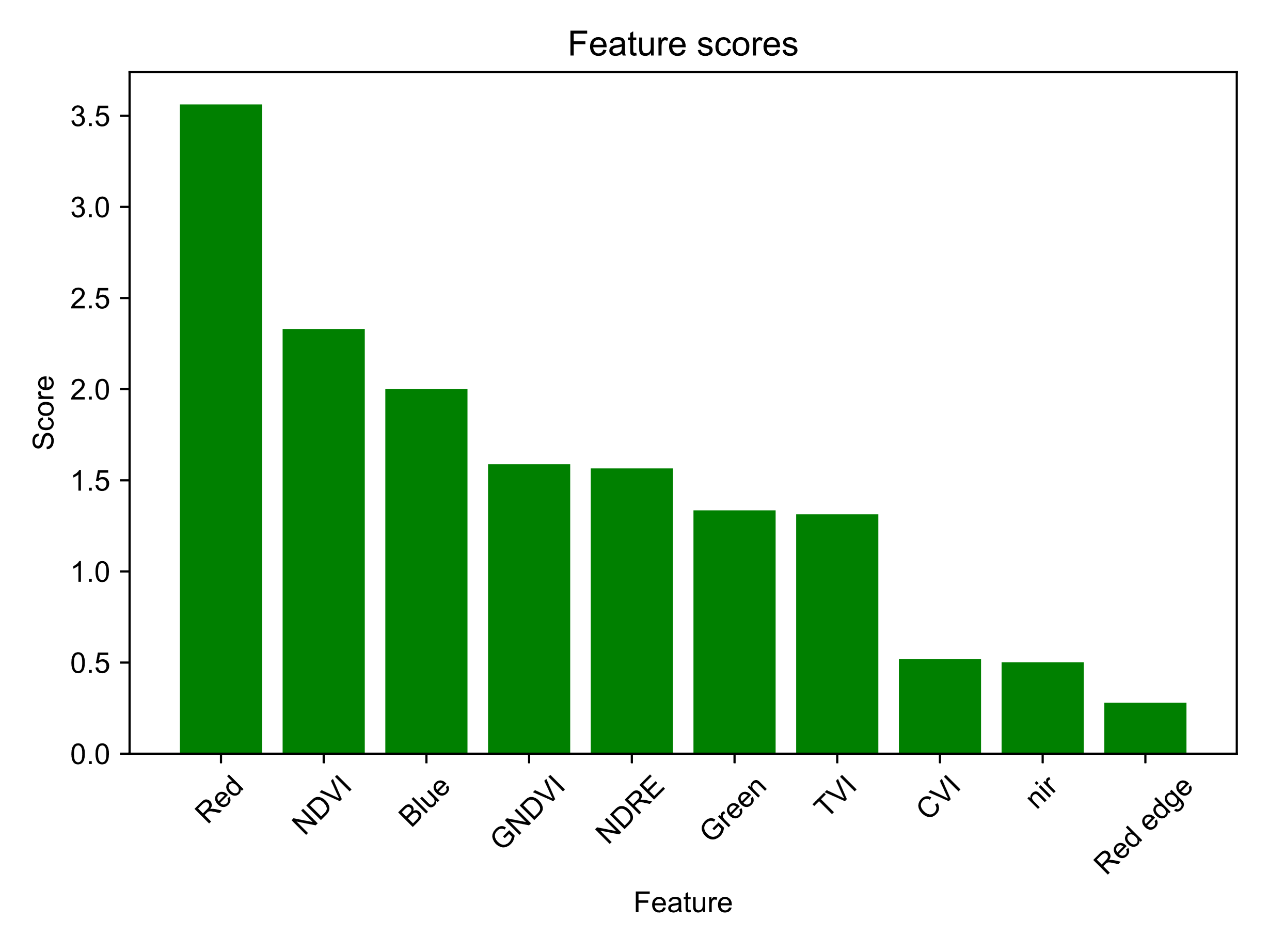


Fig. S1 Chi-square test and feature selection of the spectral vegetation indices (SVIs) and the canopy visual estimation among four sites. The bar plot of the scores of the 10 SVIs. The x-axis shows the SVIs and the y-axis shows the scores.


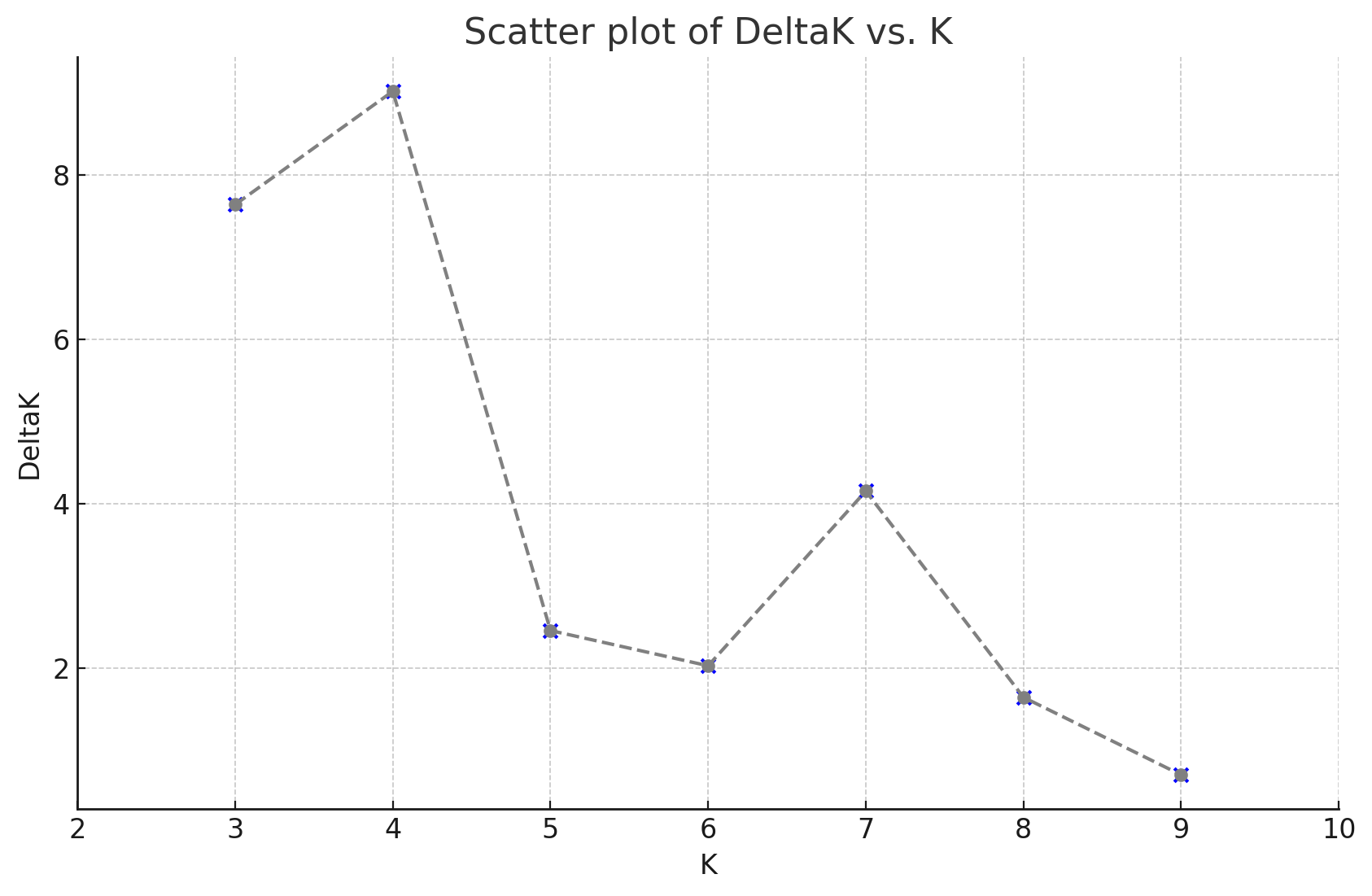


Fig. S2 Estimated ΔK for structure analysis.


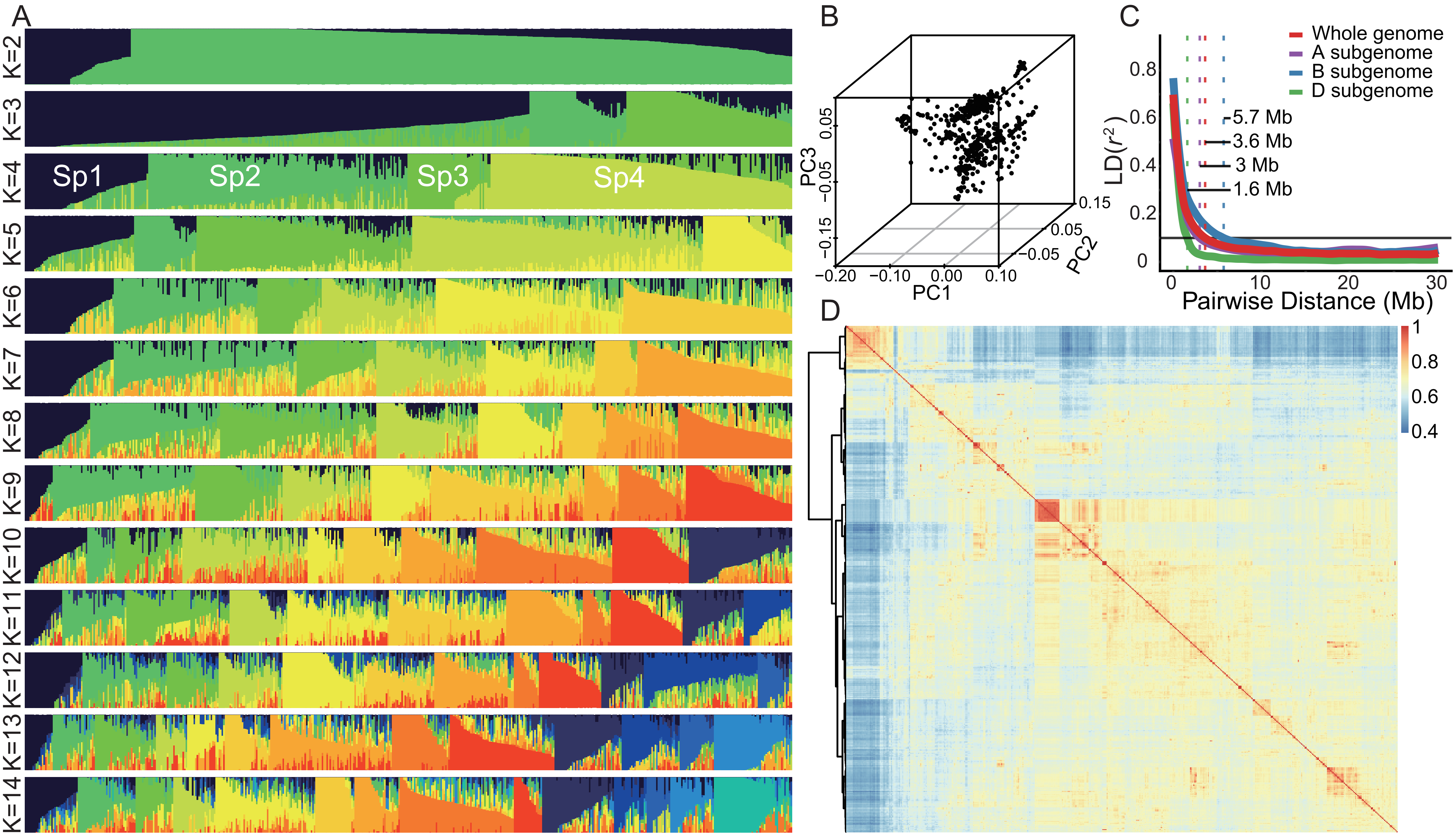


Fig. S3 Genetic structure of the diverse genotypes. (A) Population structure of the panels: defining the number of subpopulations (K) ranging from 2 to 14, the optimal population structure is K = 4. (B) Principal coordinates analysis (PCA) of wheat panels. (C) Linkage disequilibrium (LD) decay over physical distances. It portrays the pair-wise single-nucleotide polymorphism LD (*r*^2^) values as a function of inter-marker map distance within the three subgenomes. (D) The relative K-matrices. The progression from blue to red denotes a steady escalation in genetic kinship.


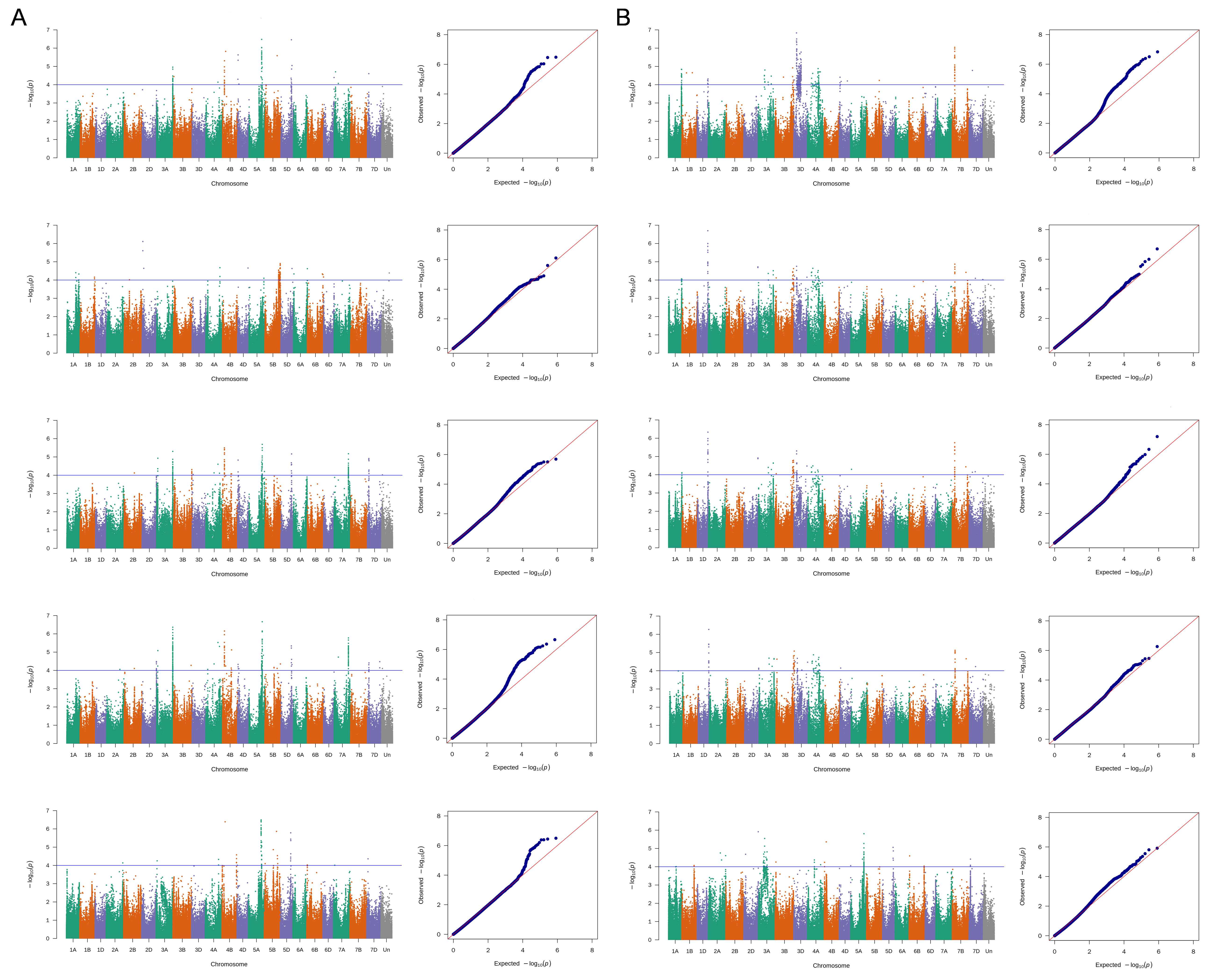

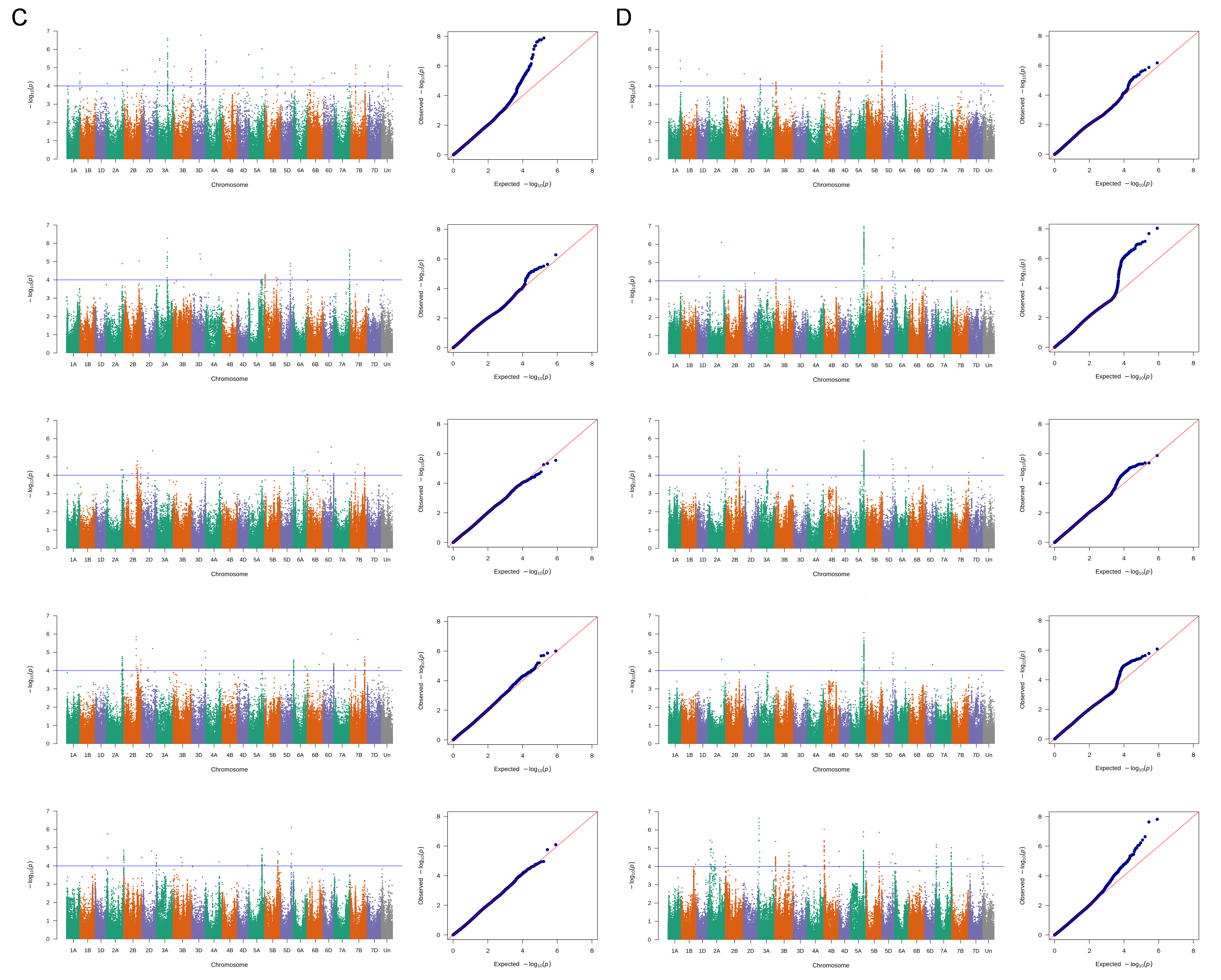


Fig. S4 Manhattan plots and Q-Q plots of BLUE, RED, NDVI, and GNDVI (from top to bottom) in SQ (A), LY (B), NY (C), and YL (D).

Fig. S5 Manhattan plots and Q-Q plots for Visual estimation (A), BLUE band (B), RED band (C), NDVI (D), and GNDVI (E) in the best linear unbiased estimate(Blue). The red dashed line indicates the threshold.

Fig. S6 Manhattan plots and Q-Q plots for Visual estimation (A), BLUE band (B), RED band (C), NDVI (D), and GNDVI (E) in the best linear unbiased estimate(Blue) of the 194 winter wheat materials carrying the *vrn-A1* allele. The red dashed line indicates the threshold.


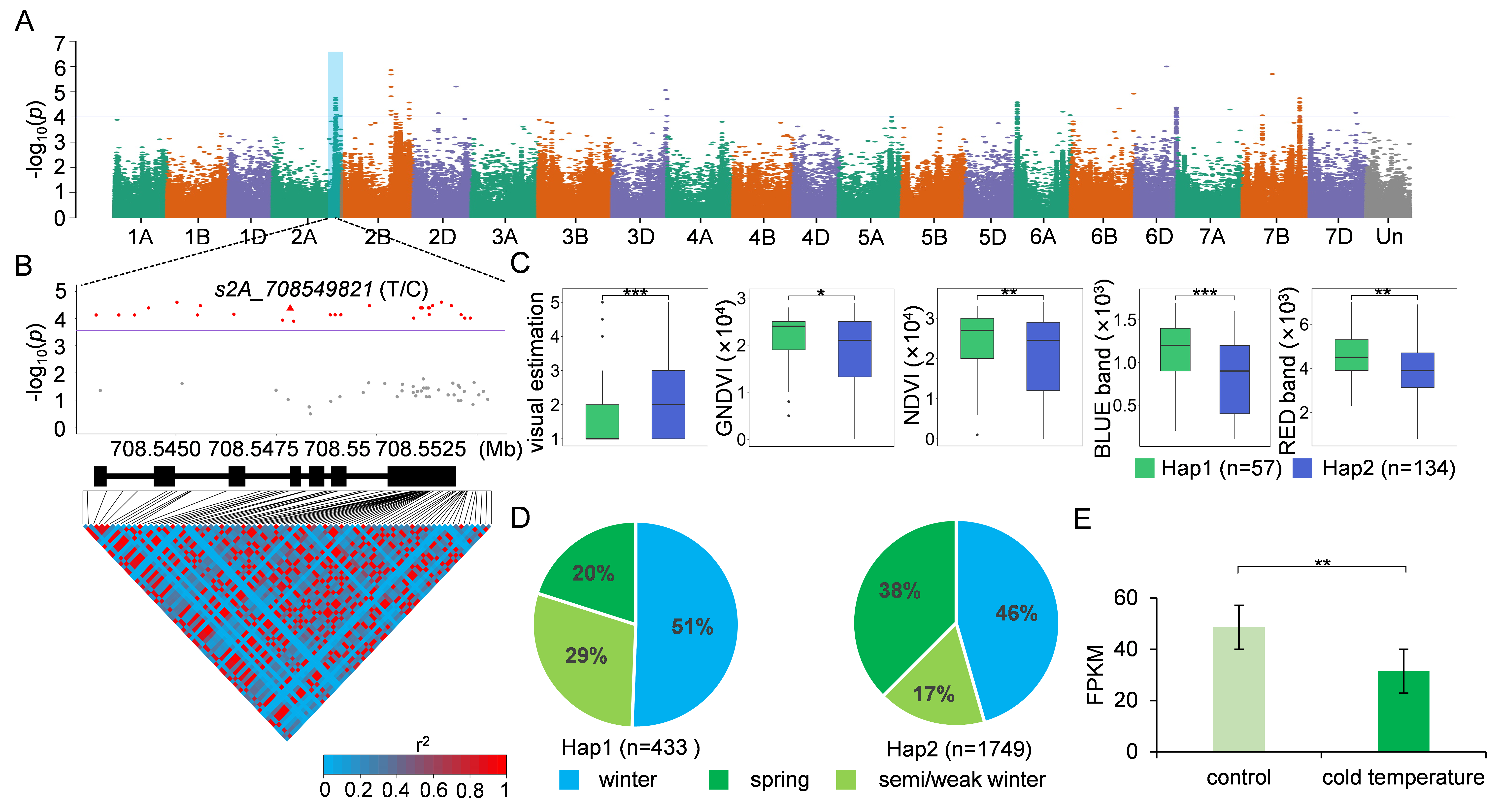


Fig. S7 Variation in *TraesCS2A03G1077800*. (A) Manhattan map displaying GNDVI in NY, with gene loci highlighted in blue on chromosome 2A and significant variation shaded in purple (−log10[*P*-value] = 4); (B) Local Manhattan plot (top) and LD heat map (bottom) surrounding *TraesCS2A03G1077800*. The red color indicates strong LD with significant variation. Red triangles represent variation in *TraesCS2A03G1077800*; (C) Comparison of visual estimation, GNDVI, NDVI, BLUE band, and RED band for the two haplotypes in SQ. *p*-values were calculated using t-tests (*, *P* < 0.05; **, *P* < 0.01; ***, *P* < 0.001; ns., not significant); (D) Percentages of haplotypes in different types of wheat; (E) Comparison of the differences in gene expression (FPKM) counts between mock (23℃) and cold temperatures (4℃) for this gene.


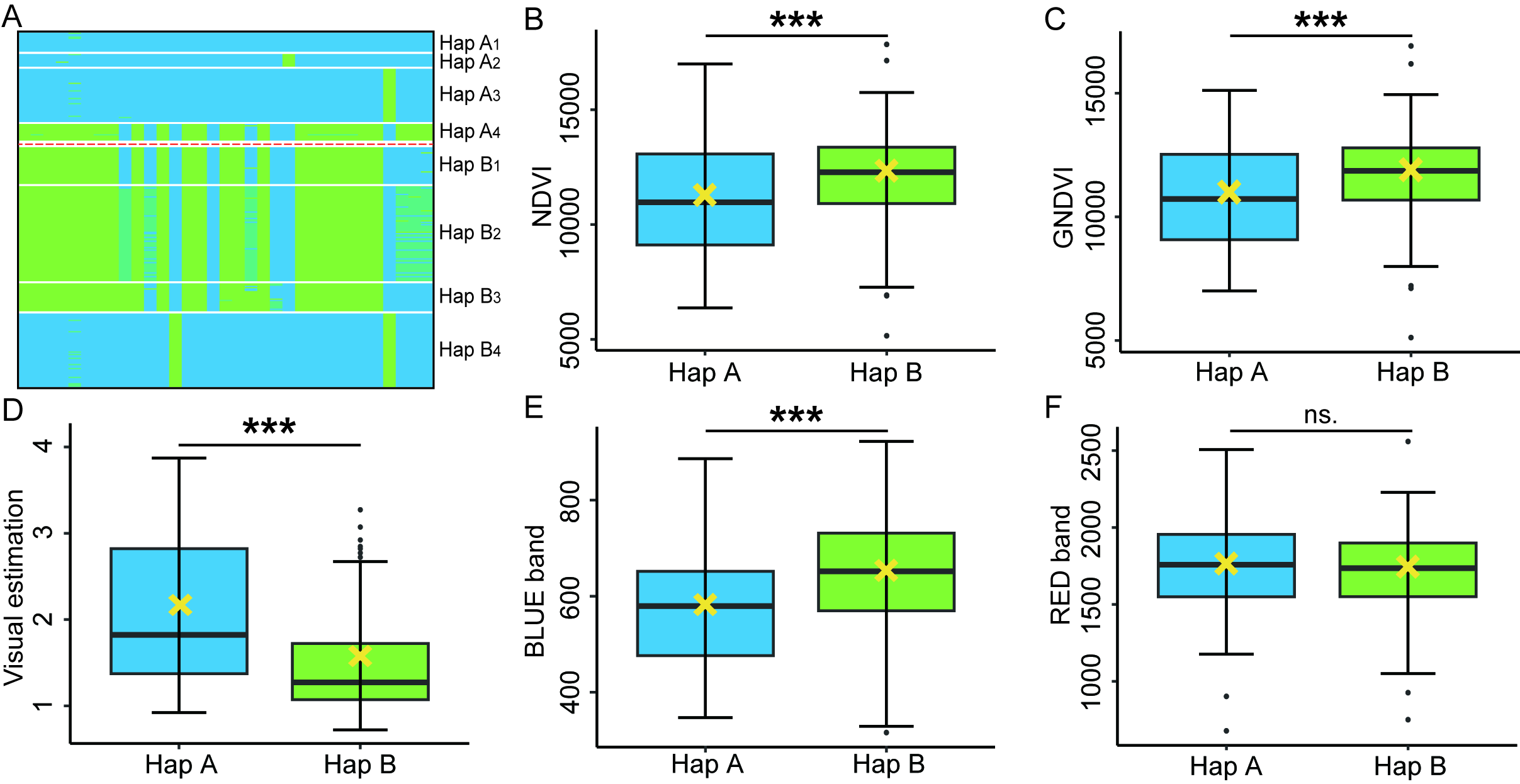


Fig. S8 The *FR-A2* haplotype is analyzed in this panel. (A)Schematic representation of the genome structure of the region of *FR-A2* upstream, gene body, and downstream. Visual estimation (D), BLUE (E), RED (F), NDVI (B), and GNDVI (C) based on the haplotypes for *FR-2* in the best linear unbiased estimate(Blue). *p*-values were calculated using t-tests (*, *P* < 0.05; **, *P* < 0.01; ***, *P* < 0.001; ns., not significant)
